# Universal Bond Models of FRP Reinforcements Externally Bonded and Near-Surface Mounted to RC Elements in Bending

**DOI:** 10.3390/ma17020493

**Published:** 2024-01-19

**Authors:** Justas Slaitas

**Affiliations:** Department of Reinforced Concrete Structures and Geotechnics, Vilnius Gediminas Technical University, Sauletekio Ave. 11, 10223 Vilnius, Lithuania; justas.slaitas@vilniustech.lt

**Keywords:** carbon, glass fibre-reinforced polymer, fracture, damage, bond model, retrofitting

## Abstract

The use of fibre-reinforced polymer materials (FRPs) for the retrofitting of reinforced concrete (RC) structures has become very popular. However, the main concern for the exploitation of FRPs is their premature debonding failure modes. This paper presents two different universal models for calculating flexed RC elements strengthened with externally bonded and near-surface mounted FRP reinforcements, which were derived by coupling principles of the fracture mechanics of solids and generally accepted assumptions. The first model allows a complete analysis of the behaviour, development, and propagation of rupture of the joint. The main advantages of the proposed model, compared to existing ones, are that it does not require additional bond shear tests to identify missing factors, and it is versatile and suitable for both externally bonded reinforcements (EBR) and near surface mounted (NSM) strengthening techniques. In addition, the concrete–FRP connection is divided into zones and the current phase and length of each zone are determined, allowing for more detailed analysis of the connection at different load stages. The proposed computational model and its derivation focus on the performance of the joint between the two cracks and the distribution of the shear stresses in that joint. The second one requires fewer computations and can be fully exploited when the joint is treated as a unit, without division. The results of the calculations have been validated using the experimental database of 77 RC beams and strengthened with externally bonded and near-surface mounted carbon fibre reinforced polymer (CFRP) and glass fibre reinforced polymer (GFRP) sheets, plates, strips, and bars taken from 13 different studies. Both the prestress force and the initial stress state before strengthening were evaluated.

## 1. Introduction

Many buildings, bridges, and other structures around the world are in bad or even dangerous condition. Such a condition is usually caused by aging, lack of maintenance, corrosion, design, or construction errors. Consequently, strengthening such structures is often necessary. Since its development in the 1980s, the use of fibre-reinforced polymer materials (FRPs) for the retrofitting of reinforced concrete structures has become very popular [1,2,3,4]. FRP materials are lighter and easier to install than traditional reinforcement materials such as concrete jackets, steel plates, etc. FRPs are used to reduce labour costs, but material costs themselves are higher. FRP materials are thin (good for aesthetics and design) and corrosion-resistant compared to steel (durability requirements) [1,5,6,7,8,9,10,11,12]. FRP systems use four common fibre types: aramide, carbon, glass, and basalt fibre [1,5,6,13,14,15]. Glass fibre-reinforced polymers (GFRPs) are the most popular type due to their low cost, but more expensive carbon fibre-reinforced polymers (CFRPs) have gained popularity due to their higher strength and tensile modulus of elasticity. In general, FRP systems can be classified into two categories: externally bonded (EBR) when FRP sheets, plates, and strips that are attached to concrete surfaces, and near-surface mounted (NSM) when a circular or rectangular FRP bar or strip is attached to the groove of the concrete surface [16,17,18,19,20,21,22,23,24,25,26].

In the case of RC structures strengthened by FRPs, seven typical failure modes can be distinguished [14]: (1) flexural failure caused by FRP rupture; (2) flexural failure caused by compression of the concrete; (3) shear failure; (4) separation of the concrete cover for tensile reinforcement; (5) interfacial debonding at the end of the FRP plate; (6) interfacial debonding caused by a flexural crack in the beam’s mid-span; and (7) interfacial debonding caused by a flexural–shear crack in the beam’s mid-span. Failure modes caused by FRP failure (mode 1) are appreciated because, in this case, all the strength of the FRP is used. However, the most common failure mode is associated with the sudden and unstable debonding of the FRP (modes 4–7) (see [14,27,28]).

The prestressing of FRPs could be a solution for using the full potential of the high tensile strength of FRPs (see [7,29,30]). Other benefits of prestressed FRPs include reduced deflection, crack width control, restored prestress losses in a prestressed concrete beam, higher concrete cracking and tensile reinforcement yield loads of strengthened beams, and stress redistribution [6,31]. However, regardless of which method of strengthening is chosen, the main concern for FRP exploitation is its premature failure mode of debonding. Since the failure of the connection between concrete and the FRP is caused by the non-full composite action (partial shear connection), choosing the most appropriate bond model is essential to obtain the actual stress–strain stage of the element. Concrete–reinforcement bond models are being developed year after year, such as several recent examples [32,33,34] in which the interaction between the FRP reinforcement and the concrete is evaluated under different environmental influences. However, when it comes to retrofitting structures, the additional reinforcement appears on the outside of the concrete, not inside it, and we have a glue bond rather than a direct bond, with a much higher potential for slipping, which ultimately leads to the premature debonding failure of the structural member. With this partial shear connection, the Euler-Bernoulli hypothesis that plane sections before bending remain plane after bending is no longer valid, and a suitable model for the assessment of the stiffness of the connection must be applied to obtain the safety and serviceability limit states.

The main types of FRP–concrete adhesive bond models were described in [35], where the most popular models were based on principles of fracture mechanics. Unfortunately, the existing models for joint assessment have a number of limitations, with the decisive factors mostly derived from FRP pull-off shear tests, which severely restricts the versatility of those models. In line with this trend, the universal methodology was developed for the calculation of flexed RC elements strengthened with externally bonded and near-surface mounted FRP reinforcements, coupling principles of the fracture mechanics of solids and generally accepted assumptions with a modified bond model proposed by Bianco et al. [36,37]. The main advantages of the proposed model compared to existing ones are that it does not require additional bond shear tests to identify missing factors, and the model is versatile and suitable for both EBR and NSM strengthening techniques. Also, the concrete–FRP connection is divided into zones, with the current stage and length of each zone being determined, which is necessary for a more detailed analysis of the connection at different stages of loading. It should be noted that the bond between the concrete and the FRP can be considered as rigid as long as the element is uncracked. However, when flexural cracks in the concrete open up, the bond is damaged, slippage occurs, and the bond breaks into smaller segments. The low flexural stiffness of the FRP reinforcement itself leads to a high probability of bond failure at the intermediate crack. The proposed computational model and its derivation focus on the performance of the joint between the two cracks and the distribution of shear stresses in that joint. The behaviour of the flexural member mentioned above is different from that of the FRP pull-off shear tests.

As a counterexample, the analytical bond model of Slaitas et al. [35,38,39,40,41,42] was used. The latter does not break down the behaviour of the bond into stages but rather takes the totality of the bond’s effects.

The numerical results are compared to the experimental results of tests on 77 RC beams with CFRP and GFRP sheets, plates, strips, and bars reinforced using the EBR or NSM methods. Experimental results were collected from various scientific publications [16,17,20,21,22,23,24,25,43,44]. Based on the obtained results, the predictive performance of the developed approach in terms of load-bearing capacity was more than sufficient with a coefficient of variation of less than 15%, which demonstrates a good level of accuracy.

## 2. Bond Model Based on the Fracture Mechanics of Solids

**Figure 1:**
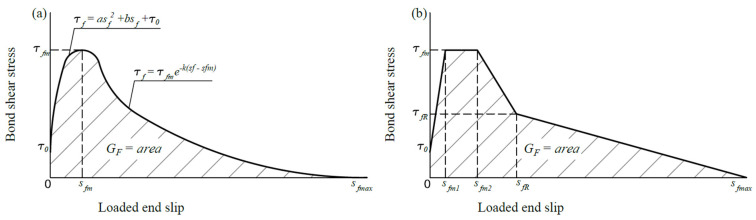
Bond shear stress–slip diagrams: (**a**) nonlinear, (**b**) multilinear.

Bond shear stress–slip relationships can be described in parabola–exponential nonlinear stress–slip diagrams (see Figure 1a) and then converted into equivalent multilinear diagrams (see Figure 1b) to facilitate integration.

The initial point of slip can be defined as a lower value of the tensile strength of concrete or an adhesive on the concrete/FRP surface.

(1) $$\tau_{0}=\min\left\{\begin{array}{l} f_{ct};\\ f_{at}, \end{array}\right.$$

where *f_at_* is the tensile strength of an adhesive on the concrete/FRP surface and *f_ct_* is the tensile strength of concrete.

**Figure 2:**
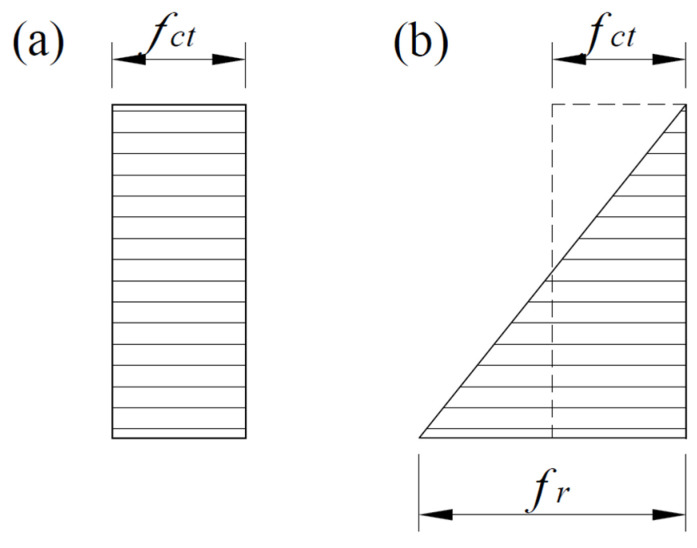
Tensile strength of concrete: (**a**) tension, (**b**) bending.

Since it is assumed that there is no slip until the concrete cracks, the starting point of the slip is determined by the concrete tensile strength. The tensile strength of concrete in the tension of elements can be calculated using the rectangular stress diagram and the triangular stress diagram in the bending of elements (see Figure 2). Thus, the concrete can be used in the pure tension expression presented in [45]:

(2) $$f_{ct}=\left\{\begin{array}{l} 0.3\sqrt[{3}]{{\left(f_{cm}-8\right)}^{2}};\;\;\;f_{cm}\leq 58\;\mathrm{MPa},\;\mathrm{tension}\\ 2.12\ln\left(1+f_{cm}/10\right);\;\;\;f_{cm}>58\;\mathrm{MPa},\;\mathrm{tension} \end{array}\right.$$

where $f_{cm}$ is the average value of the compression strength of the concrete cylinder.

When the element is in the bending zone, the triangular stress diagram is used, and the rupture module presented in [46] is better suited to the bending test result than the tensile strength proposed in EC2. Changing the triangle stress diagram to the equivalent rectangular tension of concrete is as follows:

(3) $$f_{ct}=f_{r}/2.$$

**Figure 3:**
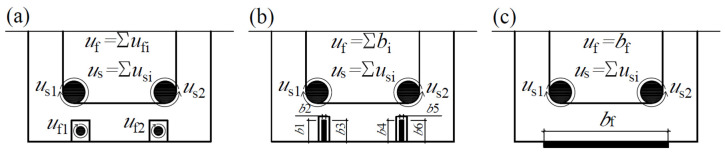
Bond perimeter of FRP (fibre reinforced polymer) and steel reinforcements: (**a**) FRP rods; (**b**) FRP strips; (**c**) EBR (externally bonded reinforcement).

According to [47], the mean maximum bond strength, i.e., the maximum tensile stress of the EBR, is limited by the concrete bond in a single (uncracked) anchorage zone.

(4) $$f_{fbm}=k_{m}k_{b}\beta_{l}\sqrt{\frac{2E_{f}}{t_{f}}f_{cm}^{2/3}},$$

where *E_f_* is the modulus of elasticity of the FRP reinforcement; shape factor *k_b_* and calibration coefficient *k_m_* were neglected and taken equal to one; and length factor $\beta_{l}$ is calculated using Equation (6). The thickness of the FRP reinforcement in EBR *t_f_* can be changed into equivalent thickness:

(5) $$t_{eq}=\frac{A_{f}}{u_{f}};$$

(6) $$\beta_{l}=\frac{l_{cr}}{2l_{b.\max}}\left(2-\frac{l_{cr}}{2l_{b.\max}}\right)\leq 1.0,$$

where *A_f_* is the area of the FRP reinforcement, *u_f_* is the bond perimeter of the FRP reinforcement (see Figure 3), and *l_cr_* is the crack spacing, the maximum bond length (*l_b._*_max_) can be estimated as follows:

(7) $$l_{b.\max}=\sqrt{\frac{E_{f}A_{f}}{2f_{ct}u_{f}}}.$$

The crack spacing *l_cr_* is calculated according to the provisions of [48]. The average crack spacing takes into account the effects of both internal and external reinforcement.

(8) $$l_{cr.m}=\frac{2f_{ct}A_{ct.eff}\xi_{b}E_{f}A_{f}}{1.25f_{ct}u_{f}\left(E_{s1}A_{s1}+\xi_{b}E_{f}A_{f}\right)},$$

where $A_{s1},E_{s1}$ are the area and modulus of elasticity of steel reinforcement; *ξ_b_* is a bond parameter (Equation (10)); and $A_{ct.eff}$ is the concrete effective area in tension.

The ultimate stress–strain state should be located at the maximum crack spacing point, which can be found from the following relation [45]:

(9) $$l_{cr}\approx 1.7l_{cr.m}=2.72\frac{A_{ct.eff}\xi_{b}E_{f}A_{f}}{u_{f}\left(E_{s1}A_{s1}+\xi_{b}E_{f}A_{f}\right)}.$$

For FRP reinforcement pull-off shear tests, the effective bond length should be used for the calculation $l_{b.eff}=\min\left(l_{b},l_{b.\max},l_{cr}\right)\approx \min\left(l_{b},l_{b.\max},8bh/30u_{f}\right)$, where *l_b_* is the bond length.

Bond parameter [48]:

(10) $$\xi_{b}=\frac{\tau_{f}E_{s1}A_{s1}u_{f}}{\tau_{s1}E_{f}A_{f}u_{s1}}=\frac{1.25E_{s1}A_{s1}u_{f}}{1.8E_{f}A_{f}u_{s1}}.$$

**Figure 4:**
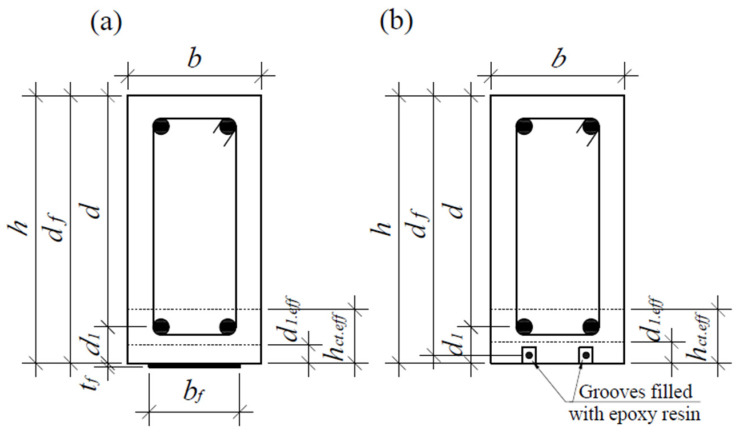
The effective area of concrete in tension (**a**) EBR (externally bonded reinforcement); (**b**) NSM (near surface mounted) [39].

The effective area of concrete in tension (see Figure 4):

(11) $$A_{ct.eff}=bh_{ct.eff}=\min\left\{\begin{array}{l} 2.5bd_{1.eff};\\ b\left(h-x_{c}\right)/3;\\ bh/2, \end{array}\right.$$

where *b* and *h* are the width and the depth of the beam; *h_cr_* is the concrete crack depth; and *d*_1.*eff*_ is the depth resultant of the internal and external reinforcements:

(12) $$d_{1.eff}=d_{1}-\frac{A_{f}\sigma_{f}\left(d_{f}-d\right)}{A_{f}\sigma_{f}+A_{s1}f_{y}}.$$

Concrete compressive strength in Equation (4) can be replaced with equivalent tensile strength:

(13) $$f_{cm}^{2/3}\approx \frac{10f_{ct}}{3}.$$

Taking into account proposed replacements, maximum tensile stress limited by bond to concrete in a single (uncracked) anchorage area is as follows:

(14) $$f_{fbm}=\beta_{l}\sqrt{\frac{20E_{f}u_{f}}{3A_{f}}f_{ct}}.$$

Bond–slip and strength depend not only on the tensile strength of the concrete, but also on the adhesion strength of the concrete/FRP surface. Therefore, concrete strength can be replaced by the shear stress at the starting point of the slip. The above assumptions allowed for expression of the maximum bond shear stress with the following equation:

(15) $$\tau_{fm}=\frac{2A_{f}f_{fbm}}{l_{cr}u_{f}}=\frac{2A_{f}\beta_{l}}{l_{cr}u_{f}}\sqrt{\frac{20E_{f}u_{f}}{3A_{f}}\tau_{0}}.$$

**Figure 5:**
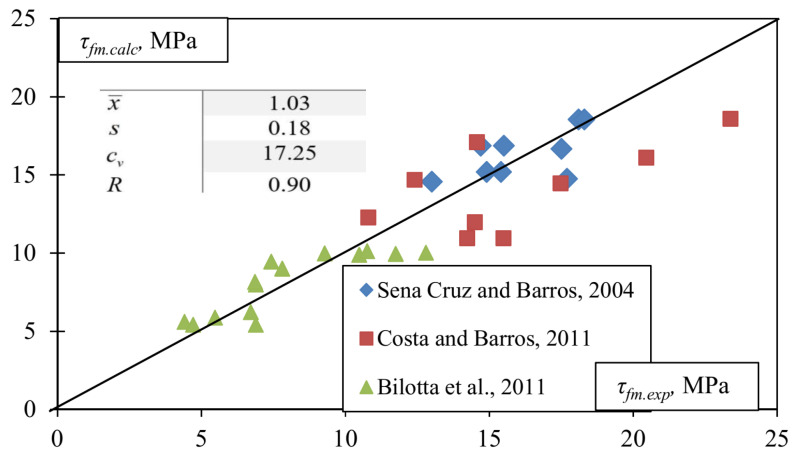
Experimental verification of maximum bond shear stress in Equation (15) (experimental data from [49,50,51]).

The comparison of experimental [49,50,51] and the numerical results of the FRP pull-off shear tests are presented in Figure 5.

Statistical parameters in Figure 5:

(16) $$\begin{array}{cc} x_{i}=\frac{\tau_{fm.\exp.i}}{\tau_{fm.calc.i}};\bar{x}=\frac{1}{n}\sum_{i=1}^{n}x_{i};s=\sqrt{\frac{1}{n-1}\sum_{i=1}^{n}{\left(x_{i}-\bar{x}\right)}^{2}};c_{v}=\frac{s}{\bar{x}}\cdot 100\%; & \\ R=\frac{\sum \left(\tau_{fm.\exp.i}-{\bar{\tau }}_{fm.\exp}\right)\left(\tau_{fm.calc.i}-{\bar{\tau }}_{fm.calc}\right)}{\sqrt{\sum {\left(\tau_{fm.\exp.i}-{\bar{\tau }}_{fm.\exp}\right)}^{2}\sum {\left(\tau_{fm.calc.i}-{\bar{\tau }}_{fm.calc}\right)}^{2}}}. & \end{array}$$

According to [52], the bond–slip is the difference between the deformation of the FRP and the concrete. The width of the crack is equivalent to double reinforcement slip. Then, if the linear shear stress–slip relation is assumed, the reinforcement slip can be expressed with the following equation (the crack width expression is derived from [45]):

(17) $$s_{f}=\frac{w_{cr}}{2}=\frac{l_{cr}}{2}\left(\varepsilon_{f.m}-\varepsilon_{ct.m}\right),$$

where the average strain difference between the FRP reinforcement and the concrete $\left(\varepsilon_{f.m}-\varepsilon_{ct.m}\right)$ can be expressed with the following well-known expression, taking into account the tension-stiffening effect of the flexural member:

(18) $$\varepsilon_{f.m}-\varepsilon_{ct.m}=\varepsilon_{f}\left(1-{\left(\frac{M_{cr}}{M_{a}}\right)}^{2}\right)+\left(\varepsilon_{f.el}-\varepsilon_{cr}\right){\left(\frac{M_{cr}}{M_{a}}\right)}^{2},$$

where *M_cr_* and *M_a_* are concrete cracking and acting bending moments respectively and $\varepsilon_{f}$ is FRP strain, the difference of FRP and tensile concrete strain between the cracks could expressed as follows:

(19) $$\varepsilon_{f.el}-\varepsilon_{cr}\approx \frac{\left(M_{a}-M_{p}\right)\left(d_{f}-0.5h\right)}{E_{c}I_{c.uncr}}-\frac{2f_{ct}}{E_{c}},$$

where *I_c.uncr_* is the second moment of area of the uncracked concrete section.

Bond–slip corresponding to maximum shear stress:

(20) $$s_{fm}\approx \frac{l_{cr}}{2}\left(\frac{\tau_{fm}l_{cr}u_{f}}{E_{f}A_{f}}\left(1-{\left(\frac{2f_{ct}E_{f}A_{f}}{E_{c}\tau_{fm}l_{cr}u_{f}}\right)}^{2}\right)+\left(\frac{\tau_{fm}l_{cr}u_{f}{\left(d_{f}-0.5h\right)}^{2}}{E_{c}I_{c.uncr}}-\frac{2f_{ct}}{E_{c}}\right){\left(\frac{2f_{ct}E_{f}A_{f}}{E_{c}\tau_{fm}l_{cr}u_{f}}\right)}^{2}\right).$$

**Figure 6:**
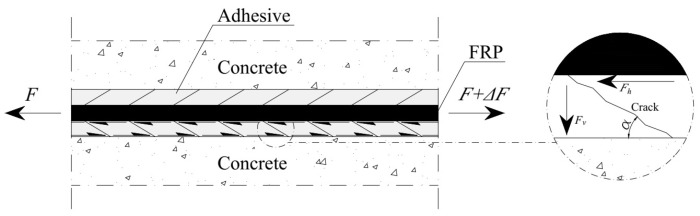
The angle between horizontal and vertical forces in the bond.

Bond shear stress at the end of the softening stage and the start of the softening–friction stage can be expressed as a sum of shear stress at the starting point of slip and bond friction stress:

(21) $$\tau_{fR}\approx \tau_{0}+\varphi \sigma_{n},$$

where φ is a coefficient of friction, φ≈0.5 can be taken, if unknown. Normal stress in the bond:

(22) $$\sigma_{n}\approx \frac{F_{v}}{u_{f}l_{cr}}=\frac{F_{h}\tan\left(\alpha \right)}{u_{f}l_{cr}}\approx \tau_{fm}\tan\left(\alpha \right),$$

where *F_v_* and *F_h_* are the vertical and horizontal forces of the bond, respectively (if unknown, the angle between them can be considered equal to $\alpha \approx 45^{0}$) (see Figure 6).

From the similarity of the triangle bond–slip at the beginning of the softening–friction phase:

(23) $$s_{fR}=\frac{s_{fm}\left(2\tau_{fm}-\tau_{fR}-\tau_{0}\right)}{\tau_{fm}-\tau_{0}}.$$

The bond shear stress at the end of the softening–friction phase leaves only the friction force between the concrete and the FRP:

(24) $$\tau_{fR-p}=\tau_{fR}-\tau_{0}.$$

At the beginning of the friction–plastic phase, the bond–slip is:

(25) $$s_{fR-p}\approx 2s_{fR}.$$

The relation between the shear stress of the bond and the slip from the beginning of the slip to the end of the softening phase can be assumed as a parabola:

(26) $$\tau_{f}\left(s_{f}\leq s_{fR}\right)=a_{f}s_{f}^{2}+b_{f}s_{f}+c_{f},$$

$\tau_{f}\left(0\right)=\tau_{0}$, then $c_{f}=\tau_{0}$. Also, there are two known points: the maximum shear stress and the end of the softening phase. The equation system is as follows:

(27) $$\left\{\begin{array}{l} \tau_{fm}-\tau_{0}=a_{f}s_{fm}^{2}+b_{f}s_{fm};\\ \tau_{fR}-\tau_{0}=a_{f}s_{fR}^{2}+b_{f}s_{fR}. \end{array}\right.$$

Solution of Equation (26):

(28) $$\left\{\begin{array}{l} a_{f}=\frac{\tau_{fm}-\tau_{0}-b_{f}s_{fm}}{s_{fm}^{2}};\\ b_{f}=\frac{\left(\tau_{fR}-\tau_{0}\right)s_{fm}^{2}-\left(\tau_{fm}-\tau_{0}\right)s_{fR}^{2}}{s_{fm}^{2}s_{fR}-s_{fR}^{2}s_{fm}}. \end{array}\right.$$

The phases of softening friction and friction plastic can be assumed to be linear, and the final energy of the fracture is as follows:

(29) $$G_{F}=\int_{0}^{s_{fR}}\tau_{f}\left(s_{f}\right)ds_{f}+\frac{2\tau_{fR}-\tau_{0}}{2}s_{fR}+\left(\tau_{fR}-\tau_{0}\right)\left(s_{f\max}-2s_{fR}\right).$$

The maximum value of bond–slip can be derived from Equation (28):

(30) $$s_{f\max}=\frac{2G_{F}-2a_{f}s_{fR}^{3}/3-b_{f}s_{fR}^{2}+s_{fR}\left(2\tau_{fR}-3\tau_{0}\right)}{2\left(\tau_{fR}-\tau_{0}\right)}.$$

Fracture energy in Equation (29) is determined from the model proposed by Bazant and Becq-Giraudon [53], which was additionally verified in [54]:

(31) $$G_{F}=2.5\alpha_{0}{\left(\frac{f_{cm}}{0.051}\right)}^{0.46}{\left(1+\frac{d_{\max}}{11.27}\right)}^{0.22}{\left(\frac{W}{C}\right)}^{-0.3},$$

where $\alpha_{0}=1$ for the rounded aggregate and $\alpha_{0}=1.44$ for the crushed aggregate, *d_max_* is the maximum aggregate size and *W*/*C* is the ratio of water to cement. If the composition of the concrete is unknown, then the fracture energy can be calculated as proposed in [47]:

(32) $$G_{F}=73f_{cm}^{0.18}.$$

The following mathematically correct replacement of the parabola–bilinear shear stress–slip diagram of the bond is presented. The elastic stage:

(33) $$\tau_{0}=f_{ct};s_{0}=0;$$

(34) $$\tau_{1}=\tau_{fR};$$

(35) $$s_{1}=\frac{\tau_{1}-\tau_{0}}{b_{f}},$$

where *b_f_* is the coefficient of the parabola equation (Equation (27)).

The hardening stage:

(36) $$\tau_{2}=\tau_{fm};$$

(37) $$s_{2}=\frac{2a_{f}s_{fm}^{3}/3+b_{f}s_{fm}^{2}+2\left(\tau_{0}-\tau_{fm}\right)s_{fm}+\left(\tau_{fm}-\tau_{0}\right)s_{1}}{\tau_{1}-\tau_{fm}}.$$

The plastic stage:

(38) $$\tau_{3}=\tau_{fm};$$

(39) $$s_{3}=\frac{2a_{f}\left(s_{fR}^{3}-s_{fm}^{3}\right)/3+b_{f}\left(s_{fR}^{2}-s_{fm}^{2}\right)+\left(2\tau_{0}-\tau_{fm}-\tau_{fR}\right)s_{fR}+2\left(\tau_{fm}-\tau_{0}\right)s_{fm}}{\tau_{fm}-\tau_{fR}}.$$

The softening stage:

(40) $$\tau_{4}=\tau_{fR};$$

(41) $$s_{4}=s_{fR}.$$

The softening–friction stage:

(42) $$\tau_{5}=\tau_{fR}-\tau_{0};s_{5}=2s_{fR}.$$

The friction–plastic stage:

(43) $$\tau_{6}=\tau_{5};s_{6}=s_{f\max}.$$

The bond shear stresses and slip of the FRP are not evenly distributed over the length of the joint, and there will be different bond shear stress–slip stages throughout the joint. As mentioned above, after concrete cracking, the concrete-FRP joint is divided into separate elements between the two cracks. It is necessary to determine the transmission length of each stage, which is limited by crack spacing, and the force generated in that part of the joint. The sum of all the forces between the two cracks will give the total ultimate force transmitted through the joint.

Governing equation for shear stress transfer [36]:

(44) $$\tau \left(x\right)=\frac{1}{J}\frac{d^{2}s\left(x\right)}{dx^{2}};$$

(45) $$J=\frac{u_{f}}{E_{f}A_{f}}+\frac{u_{f}}{E_{c}A_{ct.eff}}.$$

Shear stress in a multilinear diagram:

(46) $$\tau \left(s\right)=\left\{\begin{array}{c} \tau_{0}+\frac{\tau_{1}-\tau_{0}}{s_{1}}s;\;\;\;0\leq s\leq s_{1}\\ \tau_{1}+\frac{\tau_{2}-\tau_{1}}{s_{2}-s_{1}}\left(s-s_{1}\right);\;\;\;s_{1}<s\leq s_{2}\\ \tau_{2}=\tau_{3};\;\;\;s_{2}<s\leq s_{3}\\ \tau_{3}-\frac{\tau_{3}-\tau_{4}}{s_{4}-s_{3}}\left(s-s_{3}\right);\;\;\;s_{3}<s\leq s_{4}\\ \tau_{4}-\frac{\tau_{4}-\tau_{5}}{s_{5}-s_{4}}\left(s-s_{4}\right);\;\;\;s_{4}<s\leq s_{5}\\ \tau_{6}=\tau_{5};\;\;\;s_{5}<s\leq s_{6} \end{array}\right.$$

Bernoulli’s solution for Equations (43) and (45) will result in the bond–slip:

(47) $$s\left(x\right)=\left\{\begin{array}{c} C_{1}e^{\mu_{1}x_{e}}+C_{2}e^{-\mu_{1}x_{e}}-C_{e};\;\;\;0\leq s\leq s_{1};\;\;\;0\leq x_{e}\leq L_{tr.e}\\ C_{3}e^{\mu_{2}x_{h}}+C_{4}e^{-\mu_{2}x_{h}}-C_{h}+s_{1};\;\;\;s_{1}<s\leq s_{2};\;\;\;0\leq x_{h}\leq L_{tr.h}\\ C_{p}x_{p}^{2}+C_{5}x_{p}+s_{2};\;\;\;s_{2}<s\leq s_{3};\;\;\;0\leq x_{p}\leq L_{tr.p}\\ C_{6}\sin\left(\gamma_{1}x_{so}\right)-C_{so}\left(\cos\left(\gamma_{1}x_{so}\right)-1\right)+s_{3};\;\;\;s_{3}<s\leq s_{4};\;\;\;0\leq x_{so}\leq L_{tr.so}\\ C_{7}\sin\left(\gamma_{2}x_{fr}\right)-C_{fr}\left(\cos\left(\gamma_{2}x_{fr}\right)-1\right)+s_{4};\;\;\;s_{4}<s\leq s_{5};\;\;\;0\leq x_{fr}\leq L_{tr.fr}\\ C_{fr-p}x_{fr-p}^{2}+C_{8}x_{fr-p}+s_{5};\;\;\;s_{5}<s\leq s_{6};\;\;\;0\leq x_{fr-p}\leq L_{tr.fr-p} \end{array}\right.$$

Integration constants in the elastic stage:

(48) $$C_{1}=\frac{s_{1}+C_{e}\left(1-e^{-\mu_{1}L_{tr.e}}\right)}{2\sinh\left(\mu_{1}L_{tr.e}\right)};\;\;\;\mu_{1}=\sqrt{\frac{\tau_{1}-\tau_{0}}{s_{1}}J};\;\;\;C_{2}=C_{e}-C_{1};\;\;\;C_{e}=\frac{\tau_{1}s_{1}}{\tau_{1}-\tau_{0}}.$$

The compatibility condition:

(49) $$\frac{ds\left(x_{e}=0\right)}{dx}=\mu_{1}\left(C_{1}-C_{2}\right)=\varepsilon_{cr}.$$

The transfer length of the elastic stage:

(50) $$L_{tr.e}=\frac{1}{\mu_{1}}\ln\left(\frac{\sqrt{{\left(s_{i}+C_{e}\right)}^{2}+{\left(\varepsilon_{cr}/\mu_{1}\right)}^{2}-C_{e}^{2}}+s_{i}+C_{e}}{\varepsilon_{cr}/\mu_{1}+C_{e}}\right)\leq \frac{l_{cr}}{2}.$$

The integration constants in the hardening stage:

(51) $$C_{3}=\frac{s_{2}-s_{1}+C_{h}\left(1-e^{-\mu_{2}L_{tr.h}}\right)}{2\sinh\left(\mu_{2}L_{tr.h}\right)};\;\;\;\mu_{2}=\sqrt{\frac{\tau_{2}-\tau_{1}}{s_{2}-s_{1}}J};\;\;\;C_{4}=C_{h}-C_{3};\;\;\;C_{h}=\frac{\tau_{2}\left(s_{2}-s_{1}\right)}{\tau_{2}-\tau_{1}}.$$

The compatibility condition:

(52) $$\frac{ds\left(x_{h}=0\right)}{dx}=\mu_{2}\left(C_{3}-C_{4}\right)=\mu_{1}\left(C_{1}e^{\mu_{1}L_{tr.e}}-C_{2}e^{-\mu_{1}L_{tr.e}}\right).$$

The transfer length of the hardening stage:

(53) $$L_{tr.h}=\frac{1}{\mu_{2}}\ln\left(\frac{\sqrt{{\left(s_{i}-s_{1}+C_{h}\right)}^{2}+A_{h}^{2}-C_{h}^{2}}+s_{i}-s_{1}+C_{h}}{A_{h}+C_{h}}\right);\;\;\;A_{h}=\frac{\mu_{1}}{\mu_{2}}\left(C_{1}e^{\mu_{1}L_{tr.e}}-C_{2}e^{-\mu_{1}L_{tr.e}}\right).$$

The integration constants in the plastic stage:

(54) $$C_{p}=\frac{\tau_{3}J}{2};\;\;\;C_{5}=\frac{s_{3}-s_{2}}{L_{tr.p}}-C_{p}L_{tr.p}.$$

The compatibility condition:

(55) $$\frac{ds\left(x_{p}=0\right)}{dx}=C_{5}=\mu_{2}\left(C_{3}e^{\mu_{2}L_{tr.h}}-C_{4}e^{-\mu_{2}L_{tr.h}}\right).$$

The transfer length of the plastic stage:

(56) $$L_{tr.p}=\frac{\sqrt{A_{p}^{2}+4C_{p}\left(s_{i}-s_{2}\right)}-A_{p}}{2C_{p}};\;\;\;A_{p}=\mu_{2}\left(C_{3}e^{\mu_{2}L_{tr.h}}-C_{4}e^{-\mu_{2}L_{tr.h}}\right).$$

The integration constants in the softening stage:

(57) $$C_{6}=\frac{s_{4}-s_{3}-C_{so}\left(\cos\;\left(\gamma_{1}L_{tr.so}\right)-1\right)}{\sin\;\left(\gamma_{1}L_{tr.so}\right)};\;\;\;\gamma_{1}=\sqrt{\frac{\tau_{3}-\tau_{4}}{s_{4}-s_{3}}J};\;\;\;C_{so}=\frac{\tau_{3}\left(s_{4}-s_{3}\right)}{\tau_{3}-\tau_{4}}.$$

The compatibility condition:

(58) $$\frac{ds\left(x_{so}=0\right)}{dx}=\gamma_{1}C_{6}=2C_{p}L_{tr.p}.$$

The transfer length of the softening stage:

(59) $$L_{tr.so}=\frac{1}{\gamma_{1}}\left(\mathrm{arc}\;\sin\;\left(\frac{s_{i}-s_{3}+C_{so}}{\sqrt{A_{so}^{2}+C_{so}^{2}}}\right)-\mathrm{arc}\;\tan\;\left(\frac{C_{so}}{A_{so}}\right)\right);\;\;\;A_{so}=\frac{1}{\gamma_{1}}\left(2C_{p}L_{tr.p}+C_{5}\right).$$

The integration constants in the softening–friction stage:

(60) $$C_{7}=\frac{s_{5}-s_{4}-C_{fr}\left(\cos\left(\gamma_{2}L_{tr.fr}\right)-1\right)}{\sin\left(\gamma_{2}L_{tr.fr}\right)};\;\;\;\gamma_{2}=\sqrt{\frac{\tau_{4}-\tau_{5}}{s_{5}-s_{4}}J};\;\;\;C_{fr}=\frac{\tau_{4}\left(s_{5}-s_{4}\right)}{\tau_{4}-\tau_{5}}.$$

The compatibility condition:

(61) $$\frac{ds\left(x_{fr}=0\right)}{dx}=\gamma_{2}C_{7}=\gamma_{1}\left(C_{so}\sin\;\left(\gamma_{1}L_{tr.so}\right)+C_{6}\cos\;\left(\gamma_{1}L_{tr.so}\right)\right).$$

The transfer length of the softening–friction stage:

(62) $$\begin{array}{l} L_{tr.fr}=\frac{1}{\gamma_{2}}\left(\mathrm{arc}\;\sin\;\left(\frac{s_{i}-s_{4}+C_{fr}}{\sqrt{A_{fr}^{2}+C_{fr}^{2}}}\right)-\mathrm{arc}\;\tan\;\left(\frac{C_{fr}}{A_{fr}}\right)\right);\\ A_{fr}=\frac{\gamma_{1}}{\gamma_{2}}\left(C_{so}\sin\;\left(\gamma_{1}L_{tr.so}\right)+C_{6}\cos\;\left(\gamma_{1}L_{tr.so}\right)\right). \end{array}$$

The integration constants in the friction–plastic stage:

(63) $$C_{fr-p}=\frac{\tau_{5}J}{2};\;\;\;C_{8}=\frac{s_{6}-s_{5}}{L_{tr.fr-p}}-C_{fr-p}L_{tr.fr-p}.$$

The compatibility condition:

(64) $$\frac{ds\left(x_{fr-p}=0\right)}{dx}=C_{8}=\gamma_{2}\left(C_{fr}\sin\;\left(\gamma_{2}L_{tr.fr}\right)+C_{7}\cos\;\left(\gamma_{2}L_{tr.fr}\right)\right).$$

The transfer length of the friction–plastic stage:

(65) $$\begin{array}{l} L_{tr.fr-p}=\frac{\sqrt{A_{fr-p}^{2}+4C_{fr-p}\left(s_{i}-s_{5}\right)}+A_{fr-p}}{2C_{fr-p}};\\ A_{fr-p}=\gamma_{2}\left(C_{fr}\sin\;\left(\gamma_{2}L_{tr.fr}\right)+C_{7}\cos\;\left(\gamma_{2}L_{tr.fr}\right)\right). \end{array}$$

The resisting forces:

(66) $$F_{i}=A_{f}E_{f}{\left.\frac{ds\left(x_{i}\right)}{dx}\right|}_{0}^{L_{tr.i}}.$$

(67) $$\left\{\begin{array}{l} F_{e}=E_{f}A_{f}\mu_{1}\left(C_{1}\left(e^{\mu_{1}L_{tr.e}}-1\right)-C_{2}\left(e^{-\mu_{1}L_{tr.e}}-1\right)\right);\\ F_{h}=E_{f}A_{f}\mu_{2}\left(C_{3}\left(e^{\mu_{2}L_{tr.h}}-1\right)-C_{4}\left(e^{-\mu_{2}L_{tr.h}}-1\right)\right);\\ F_{p}=E_{f}A_{f}2C_{p}L_{tr.p};\\ F_{so}=E_{f}A_{f}\gamma_{1}\left(C_{so}\sin\;\left(\gamma_{1}L_{tr.so}\right)+C_{6}\left(\cos\;\left(\gamma_{1}L_{tr.so}\right)-1\right)\right);\\ F_{fr}=E_{f}A_{f}\gamma_{2}\left(C_{fr}\sin\;\left(\gamma_{2}L_{tr.fr}\right)+C_{7}\left(\cos\;\left(\gamma_{2}L_{tr.fr}\right)-1\right)\right);\\ F_{fr-p}=E_{f}A_{f}2C_{fr-p}L_{tr.fr-p}. \end{array}\right.$$

The transfer lengths of the last part to the loaded end of the bond–slip, when the acting force is in different stages:

(68) $$\left\{\begin{array}{l} x_{e}=\frac{1}{\mu_{1}}\ln\left(\frac{\sqrt{B_{e}^{2}+4C_{1}C_{2}}+B_{e}}{2C_{1}}\right);\;\;\;F_{a}\leq F_{e}\\ B_{e}=\frac{F_{a}}{E_{f}A_{f}\mu_{1}}+C_{1}-C_{2};\;\;\;\\ x_{h}=\frac{1}{\mu_{2}}\ln\left(\frac{\sqrt{B_{h}^{2}+4C_{3}C_{4}}+B_{h}}{2C_{3}}\right);\;\;\;0<F_{a}-F_{e}\leq F_{h}\\ B_{h}=\frac{F_{a}-F_{e}}{E_{f}A_{f}\mu_{2}}+C_{3}-C_{4};\\ x_{p}=\frac{F_{a}-F_{e}-F_{h}}{E_{f}A_{f}2C_{p}};\;\;\;0<F_{a}-F_{e}-F_{h}\leq F_{p}\\ x_{so}=\frac{1}{\gamma_{1}}\left(\mathrm{arc}\;\sin\;\left(\frac{B_{so}+C_{6}}{\sqrt{C_{so}^{2}+C_{6}^{2}}}\right)-\mathrm{arc}\;\tan\;\left(\frac{C_{6}}{C_{so}}\right)\right);\;\;\;0<F_{a}-\sum_{0}^{F_{p}}F_{i}\leq F_{so}\\ B_{so}=\frac{F_{a}-\sum_{0}^{F_{p}}F_{i}}{E_{f}A_{f}\gamma_{1}};\\ x_{fr}=\frac{1}{\gamma_{2}}\left(\mathrm{arc}\;\sin\;\left(\frac{B_{fr}+C_{7}}{\sqrt{C_{fr}^{2}+C_{7}^{2}}}\right)-\mathrm{arc}\;\tan\;\left(\frac{C_{7}}{C_{fr}}\right)\right);\;\;\;0<F_{a}-\sum_{0}^{F_{so}}F_{i}\leq F_{fr}\\ B_{fr}=\frac{F_{a}-\sum_{0}^{F_{so}}F_{i}}{E_{f}A_{f}\gamma_{2}};\\ x_{fr-p}=\frac{F_{a}-\sum_{0}^{F_{fr}}F_{i}}{E_{f}A_{f}2C_{fr-p}};\;\;\;0<F_{a}-\sum_{0}^{F_{fr}}F_{i}\leq F_{fr-p} \end{array}\right.$$

The overall resisting force:

(69) $$F_{b}=F_{e}+F_{h}+F_{p}+F_{so}+F_{fr}+F_{fr-p}.$$

The acting force:

(70) $$F_{a}=E_{f}A_{f}\varepsilon_{f}.$$

The bond stiffness reduction factor:

(71) $$\psi_{f}=\frac{F_{b}}{F_{a}}\leq 1.0.$$

The bond stiffness reduction factor in Equation (70) could be used for the assessment of the ultimate and serviceability limit states. The assessment of the ultimate limit state of strengthened RC members in the bending zone is presented in the fourth chapter.

## 3. Bond Model Based on Built-Up Bars Theory

The second model for comparison is the fully analytical bond model proposed by Slaitas et al. [35,38,39,40,41,55]. In this model, the bond between the concrete and the FRP is considered as a single unit between the two cracks, without breaking it down into stages or individual sections. The object of consideration is the joint of two elements: concrete and FRP reinforcement. The analytical built-up bars solution of the bond shear force depends on the load conditions of the element. The following is the solution when the simply supported element is loaded with two concentrated forces *F*/2 [35]:

(72) $$T\left(z\right)=\frac{Faz}{\beta EI}\left(1-\frac{\cos\;h\left(\sqrt{\alpha \beta }\left(0.5L-L_{s}\right)\right)}{\sqrt{\alpha \beta }z\cos\;h\left(0.5\sqrt{\alpha \beta }L\right)}\sin\;h\left(\sqrt{\alpha \beta }z\right)\right).$$

The first member of Equation (71) represents the shear force of two rigidly connected members and the second, in brackets, is the contact stiffness reduction factor *ψ_f_*. For the beam loaded with two concentrated forces in the pure bending zone, it will be as follows [35]:

(73) $$\psi_{f}=1-\frac{\cos\;h\left(\sqrt{\alpha \beta }\left(0.5L-L_{s}\right)\right)}{\sqrt{\alpha \beta }L_{s}\cos\;h\left(0.5\sqrt{\alpha \beta }L\right)}\sin\;h\left(\sqrt{\alpha \beta }L_{s}\right);$$

(74) $$\alpha =\frac{G_{eff}u_{f}}{a};\;\;\;\beta =\frac{1}{E_{f}A_{f}}+\frac{1}{E_{c}A_{c.eff}}+\frac{a^{2}}{EI},$$

where *u_f_* is the width of the FRP to the concrete bond (or the perimeter in the case of NSM FRP bars), *E_f_A_f_* is the axial stiffness of the FRP, *E_c_A_c.eff_* is the axial stiffness of the cracked concrete section, and *EI* is the composite flexural stiffness of two elements:

(75) $$EI=E_{c}I_{c.eff}+E_{f}I_{f}+\frac{E_{c}A_{c.eff}E_{f}A_{f}a^{2}}{E_{c}A_{c.eff}+E_{f}A_{f}}.$$

**Figure 7:**
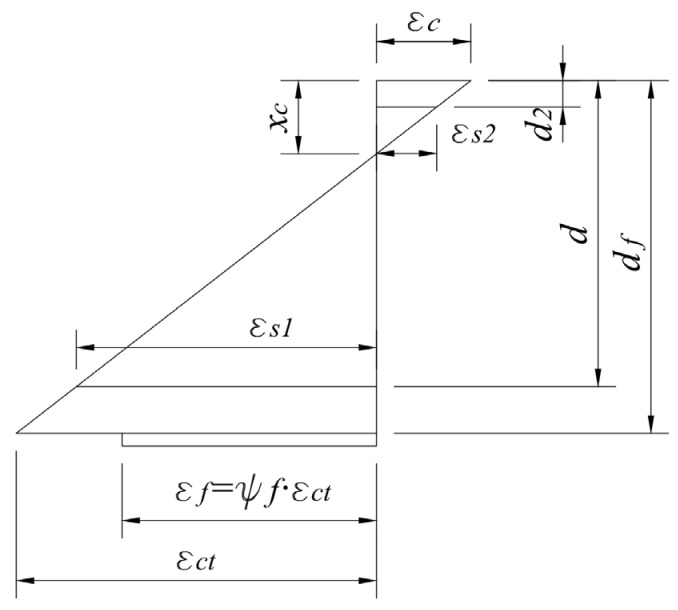
Strain profile [41].

The fully analytical solution of the effective shear modulus of the concrete and the FRP [35]:

(76) $$G_{eff}=\frac{G_{c}t_{eff}\left(E_{c}A_{ct.eff}+E_{f}A_{f}\right)}{L_{cr}^{2}u_{f}G_{c}+2t_{c}\left(E_{c}A_{ct.eff}+E_{f}A_{f}\right)},$$

where *t_c_* ≈ *a* and *t_eff_* ≈ *t_a_*, while the adhesive layer is completely deformed together with the FRP reinforcement. The adhesive layer recommended by the manufacturers is between 1–4 mm, but for safety reasons, it should be taken equal to 1 mm. *L_cr_* is the spacing of the cracks and *G_c_* is the shear modulus of the concrete. The graphic illustration of the strengthened member’s strain profile of the strengthened member taking into account a concrete–FRP partial shear connection is presented in Figure 7.

The above factors allow for evaluation of the effect of the concrete–FRP partial shear connection to the behaviour of the strengthened RC members.

## 4. Load-Bearing Capacity

**Figure 8:**
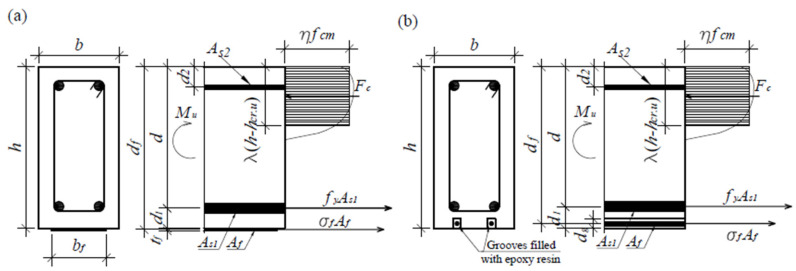
State of stress in RC beam strengthened with: (**a**) EB FRP reinforcement, (**b**) NSM FRP reinforcement [38,39].

When the load of the FRP-strengthened RC element is close to its final value, the strain of the tensile steel reinforcement element exceeds the yield strength in most cases, resulting in large plastic deformations. Thus, the tensile zone above the crack can be ignored, and the final depth of the element can be evaluated as shown in Figure 8, using an equivalent rectangular concrete compression stress diagram.

The FRP stress is not known, but it is possible to achieve the balance between the final crack depth and the FRP stress iteratively. In this way, the ultimate depth of crack can be evaluated using the following equation:

(77) $$h_{cr.u.i}=h-\frac{A_{s1}f_{y}+A_{f}\sigma_{f.i}}{\eta \lambda f_{cm}b},$$

where *f_y_* and *f_cm_* are the yield strength of the tensile steel reinforcement and the mean value of the compressive strength of concreate, respectively, *η* and *λ* are the reduction factors of the compressive strength of concrete and the compressive zone height, respectively (following [45]: *η* = 1.0, *λ* = 0.8 for concrete strength *f_ck_* < 50 MPa). The FRP stress of the *i*th step can be found assuming the linear elastic stress–strain relationship, but it must be lower than the design strength:

(78) $$\sigma_{f.i}=E_{f}\left(\varepsilon_{f.i}+\varepsilon_{p}-\varepsilon_{0}\right)\leq f_{f},$$

where $\varepsilon_{p}$ is the FRP prestressing strain, *ε*_0_ is the initial strain at the FRP level, and $\varepsilon_{f.i}$ is a strain at the FRP level neglecting the prestressing and initial strains:

(79) $$\varepsilon_{f.i}=\varepsilon_{cu}\frac{d_{f}-h+h_{cr.u.i}}{h-h_{cr.u.i}}.$$

According to [45], the ultimate strain of compressive concrete *ε_cu_* can be taken as 3.5‰ when *f_ck_* < 50 MPa. Repeat iterations until the equilibrium condition is reached:

(80) $$\sigma_{f.n}\approx \sigma_{f.n-1}.$$

The actual reduction coefficients of the concrete compressive zone stress diagram can be determined using the modified technique proposed by Dulinskas et al. [38,39,56].

Subsequently, the load-carrying capacity of the strengthened member can be expressed as follows:

(81) $$M_{u}=A_{s1}f_{y}\left(d-\frac{\lambda \left(h-h_{cr.u}\right)}{2}\right)+A_{f}E_{f}\psi_{f}\left(\varepsilon_{f}+\varepsilon_{p}-\varepsilon_{0}\right)\left(d_{f}-\frac{\lambda \left(h-h_{cr.u}\right)}{2}\right).$$

The methodology proposed in this chapter shows how to determine the ultimate crack depth, the stress–strain state in the element, and the load-carrying capacity using the correct bond model (which was proposed in previous chapters).

## 5. Validation of the Results

A number of RC beams tested by different researchers using carbon fibre-reinforced polymer (CFRP) and glass fibre-reinforced polymer (GFRP) sheets, plates, strips, and rods as retrofitting materials were analysed in a comparison of the numerical and experimental results of the load-carrying capacity. In total, experimental data from 77 different beams from 13 different studies were used for the analyses. All beams were tested using four-point bending tests. The properties of the beams and strengthening materials varied a lot, also, parts of the beams were strengthened with prestressed FRP reinforcements (*σ_p_* is FRP prestressing stress), as presented in Table 1.

**Figure 9:**
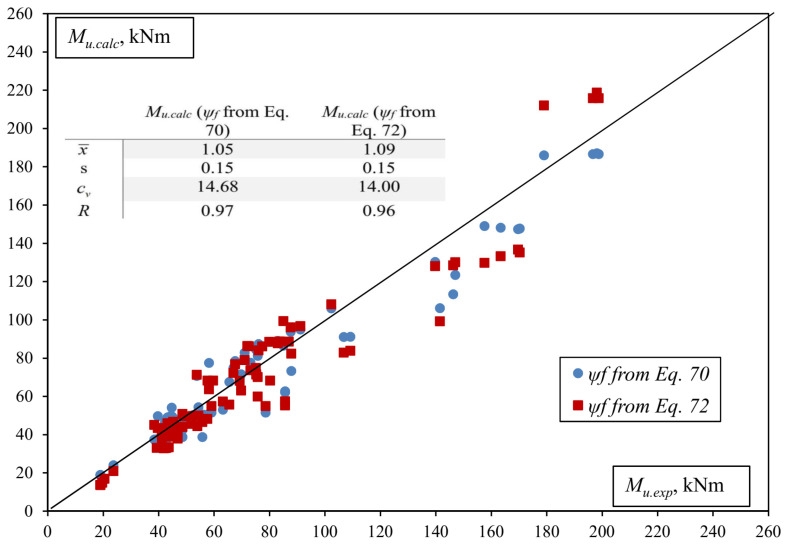
The comparison of experimental and numerical load-bearing capacity.

The comparison of the experimental and numerical load-carrying capacity results is shown in Figure 9.

Statistical parameters in Figure 9:

(82) $$x_{i}=\frac{M_{u.\exp.i}}{M_{u.calc.i}};\;\;\;\bar{x}=\frac{1}{n}\sum_{i=1}^{n}x_{i};\;\;\;s=\sqrt{\frac{1}{n-1}\sum_{i=1}^{n}{\left(x_{i}-\bar{x}\right)}^{2}};\;\;\;c_{v}=\frac{s}{\bar{x}}\cdot 100\%;\;R=\frac{\sum \left(M_{u.\exp.i}-{\bar{M}}_{u.\exp}\right)\left(M_{u.calc.i}-{\bar{M}}_{u.calc}\right)}{\sqrt{\sum {\left(M_{u.\exp.i}-{\bar{M}}_{u.\exp}\right)}^{2}\sum {\left(M_{u.calc.i}-{\bar{M}}_{u.calc}\right)}^{2}}}.$$

The results of the calculation are very similar in both cases, and it is safe to say that the carrying capacity has been calculated very accurately with a small mean error (5% and 9%), a small random error (0.15 in both cases), and a high correlation (0.97 and 0.96). It should be noted that without considering the non-composite action (partial shear connection) between the concrete and the FRP reinforcement, some of the beams will collapse after reaching only about 50% of the design capacity, which is critically unsafe, whereas the FRP–concrete bond models proposed in this paper avoid this completely.

## 6. Conclusions

1. In this paper, two different universal models are presented for the assessment of the bond between concrete and FRPs, both of which assess the behaviour of the bond between two cracks. The first model is based on the fracture mechanics of solids, distinguishing different stages of failure development and distribution over the length of the joint. The second one is the fully analytical model based on the built-up bars theory, considering the joint as a single unit.
2. In both cases, the load-bearing capacity of the member’s normal section is determined very accurately with a low mean error (5% and 9%), a low random error (0.15 in both cases), and a high correlation (0.97 or 0.96). The results of the calculation have been validated with 77 beam tests carried out by different researchers. The beams were strengthened using both EBR and NSM methods, with strong variations in performance. Both the prestress force and the initial stress state before strengthening were evaluated.
3. The first approach, based on the fracture mechanics of solids, has advantages over the second approach in that it allows for a complete analysis of the behaviour of the joint, the development, and the propagation of rupture. However, the second approach is well suited to the calculation of the load-bearing capacity, requires much less computation, and can be fully exploited where it is sufficient to treat the joint as a unit, without subdividing.
4. The most common description of a concrete–FRP bond found in the literature is based on some specific testing, or it is greatly simplified, resulting in a number of limitations in the application and a number of aspects that are not assessed. The significance of this paper lies in the fact that the proposed models are universal, not tied to specific tests, suitable for different strengthening methods, do not use any major simplifications, and the only limitation is the normal section of the bending element.

## Figures and Tables

**Table 1:** The properties of the analysed beams (EBR is externally bonded reinforcement, NSM is near surface mounted reinforcement).

Ref.	*A_s_*_1_/*bd* (%)	*f_y_* (MPa)	*A_f_*/*bd_f_* (%)	*f_f_* (MPa)	*E_f_* (GPa)	*σ_p_* (MPa)	EBR/NSM
[16]	0.85	400	0.11	3100	165	1000	EBR
[16]	0.85	400	0.13–0.14	2068	131	0–1000	NSM
[17]	0.40	426	0.04–0.12	2453–3479	165–230	0	EBR
[17]	0.40	426	0.04–0.11	1878–2453	121–165	0	NSM
[43]	0.45	436	0.04–0.22	1500–2483	100–167	0	NSM
[25]	0.29–1.19	466–501	0.08	2850	165	0–1323	EBR
[44]	0.50–0.75	525–531	0.11	3263	251	0	EBR
[23]	0.58	545	0.12–0.26	1350–2350	64–170	0	NSM
[24]	0.58	540	0.13–0.26	1350–2500	64–170	0	NSM
[20]	0.77	475	0.08	2167	130	0–1241	NSM
[21]	0.54–0.94	730	0.16–0.24	2740	159	0	NSM
[22]	0.39	585	0.06	1922	164	0–823	NSM
[39]	0.68–1.13	318–569	0.08–0.32	2334–4800	213–230	0–120	EBR

## Data Availability

Data are contained within the article.
